# Novel Microbiological and Spatial Statistical Methods to Improve Strength of Epidemiological Evidence in a Community-Wide Waterborne Outbreak

**DOI:** 10.1371/journal.pone.0104713

**Published:** 2014-08-22

**Authors:** Katri Jalava, Hanna Rintala, Jukka Ollgren, Leena Maunula, Vicente Gomez-Alvarez, Joana Revez, Marja Palander, Jenni Antikainen, Ari Kauppinen, Pia Räsänen, Sallamaari Siponen, Outi Nyholm, Aino Kyyhkynen, Sirpa Hakkarainen, Juhani Merentie, Martti Pärnänen, Raisa Loginov, Hodon Ryu, Markku Kuusi, Anja Siitonen, Ilkka Miettinen, Jorge W. Santo Domingo, Marja-Liisa Hänninen, Tarja Pitkänen

**Affiliations:** 1 Department of Infectious Disease Surveillance and Control, National Institute for Health and Welfare, Helsinki, Finland; 2 Siilinjärvi municipality, Finland; 3 Department of Food Hygiene and Environmental Health, University of Helsinki, Helskinki, Finland; 4 Office of Research and Development, United States Environmental Protection Agency, Cincinnati, Ohio, United States of America; 5 Laboratory HUSLAB, Helsinki University Hospital, Helsinki, Finland; 6 Department of Environmental Health, National Institute for Health and Welfare, Kuopio, Finland; The Australian National University, Australia

## Abstract

Failures in the drinking water distribution system cause gastrointestinal outbreaks with multiple pathogens. A water distribution pipe breakage caused a community-wide waterborne outbreak in Vuorela, Finland, July 2012. We investigated this outbreak with advanced epidemiological and microbiological methods. A total of 473/2931 inhabitants (16%) responded to a web-based questionnaire. Water and patient samples were subjected to analysis of multiple microbial targets, molecular typing and microbial community analysis. Spatial analysis on the water distribution network was done and we applied a spatial logistic regression model. The course of the illness was mild. Drinking untreated tap water from the defined outbreak area was significantly associated with illness (RR 5.6, 95% CI 1.9–16.4) increasing in a dose response manner. The closer a person lived to the water distribution breakage point, the higher the risk of becoming ill. Sapovirus, enterovirus, single *Campylobacter jejuni* and EHEC O157:H7 findings as well as virulence genes for EPEC, EAEC and EHEC pathogroups were detected by molecular or culture methods from the faecal samples of the patients. EPEC, EAEC and EHEC virulence genes and faecal indicator bacteria were also detected in water samples. Microbial community sequencing of contaminated tap water revealed abundance of *Arcobacter* species. The polyphasic approach improved the understanding of the source of the infections, and aided to define the extent and magnitude of this outbreak.

## Introduction

Community-wide waterborne outbreaks are characterized by a large number of exposed people with high attack rates [1]–[6]. Waterborne outbreaks are frequently associated with large number of symptomatic cases in a point source manner. Such outbreaks may be caused by a failure in the drinking water distribution system [1], [3], [4] or water treatment breakthrough of contaminating agents due to heavy rainfall or other excess weather conditions [2], [5]. The water distribution system can be potentially contaminated with multiple pathogens during a relatively short period of time as the result of intrusion from surface or waste water [7]. Indeed, waterborne outbreaks with multiple causative organisms, e.g. *Campylobacter* spp., norovirus-like organisms, *Shigella* and enterohaemorrhagic *Escherichia coli* (EHEC) have been described [1], [3], [4], [6], [8], [9]. In particular, when norovirus and sapovirus types are implicated in large scale waterborne outbreaks, this strongly indicates drinking water distribution system contamination by a human faecal sources [10], [11]. Sapovirus usually causes sporadic infections [12] but has been isolated from cases of waterborne outbreaks [12], [13].

Waterborne outbreaks may be classified according to the level of evidence indicating that the drinking water was the cause of the outbreak. Evidence may be found by microbiological and/or epidemiological studies and the level of evidence can be assessed according to standardized criteria [14]. During the years 1998–2009 there have been 3–10 waterborne outbreaks in Finland annually and the outbreaks have typically been detected in small community groundwater plants with fewer than 500 consumers [7]. The implicated technical failures for the groundwater contamination in Finland have been flooding and surface run-off caused by heavy rains or rapid melting of snow. Also intrusion of contaminated water and cross-connections in the water distribution system play important role as a cause of Finnish waterborne outbreaks [15], [16]. Most common causative microbes have been norovirus and *Campylobacter* [7].

In waterborne outbreak investigations, the delay between environmental investigation and the original presence of the pathogen within a water body has often hampered the detection of causative microbiological agents [15]. Special methods suitable for concentration of large water volumes, such as the use of large diameter membrane filters or ultrafiltration apparatus have been developed to increase the sensitivity of microbial detection in dilute water samples [17]–[19]. For the identification of a faecal contamination source, various detection methods targeting specific pathogens and microbial water quality indicators [15] and faecal source host specific molecular assays are available [20]. The use of RNA-based methods may not only help detecting but also identify active and thus potentially infective pathogens, providing a better estimate of public health risks than current DNA-based methods [21]. Additionally, next generation sequencing (NGS) can provide a path towards detecting multiple microbial taxa within complex microbial communities including rare members [17]. The high number of reads per sample in NGS applications also enables the use of these techniques for source tracking and identification of the transmission route of an outbreak [22].

Epidemiological cohort or case control studies may be applied in drinking water outbreaks due to the point source nature of the contamination and a well-defined population [3], [11], [23]. Web-based questionnaires are now increasingly used in outbreak investigations [24], especially in large and/or widely spread outbreaks [25]. Additionally, novel spatial methods have shown potential to define the source and location of the outbreak [26]–[28]. Modelling may be used to describe the person-to-person transmission or infection spreading from the environment [28]. Spatial variation in disease incidence has been studied in detail [27]. Also distance from water tanks has been evaluated by statistical methods with respect to diarrhoeal incidence [26].

### The outbreak

The main water pipe was accidentally broken on 4^th^ July, 2012 during road construction work in Vuorela, a community of 3000 inhabitants within the municipality of Siilinjärvi in Eastern Finland. The pipe breakage caused the contents of the upper drinking water storage reservoir to leak into the road construction pit. The pipe breakage was fixed within 14 hours, flushed and quality of the water was shown to fulfill the hygienic quality criteria. On the 16^th^ July, the local environmental health authorities were informed by the health care centre of an excess number of patients with gastrointestinal symptoms. Given the recent pipe break in the area, a waterborne outbreak was suspected. The local outbreak control team of health and environmental authorities and waterworks personnel was activated, national outbreak awareness team was informed and consulted. On the following day, the results from tap water samples revealed faecal contamination of the water further confirming the waterborne nature of the outbreak. An immediate boil water notice was issued on 17^th^ July and collection of patient and drinking water samples was initiated. The water distribution pipeline was subsequently flushed, the water storage reservoir was cleaned and the whole distribution system was disinfected with chlorine. The boil water notice was cancelled when the outbreak was declared over on 3^rd^ August 2012.

The aim of our study was to reveal the role of contaminated water as a cause of a community wide outbreak detected in a small Finnish municipality during July 2012. We used a polyphasic approach carrying out advanced epidemiological, microbiological and environmental investigation to verify the source and scale of the outbreak. We included advanced and novel statistical, epidemiological and molecular microbiology methods, not previously applied to outbreak investigations according to our knowledge.

## Materials and Methods

This study was part of public health response. According to Finnish legislation, no ethical approval is needed in this type of response. This study and related sampling and modelling were part of an official waterborne outbreak investigation, which is on the responsibility of municipal health and public health authorities (many of the coauthors are representatives of respective authorities).

### Epidemiological investigation

#### Data collection

The contaminated part of the water distribution system provides drinking water for the area of Vuorela and Toivala in the municipality of Siilinjärvi. The total population of this area was 5934 and the exposed population who were served by the contaminated water distribution system in the defined outbreak area was 2931 persons (source population). The age, sex and living coordinates for the population were obtained from the National Population Register. A case was defined as a person staying or living in the Vuorela area during July 2012 with diarrhoea or two of the following symptoms: nausea, vomiting, stomach ache or fever. We excluded persons who were absent from the outbreak area during the whole study period and those who travelled abroad during July 2012. Based on the geographical coordinates we also determined the number of households or blocks of houses with unique water delivery points in the affected area.

#### Study design

A retrospective cohort study was conducted. A web based questionnaire was designed to define the extent and cause of the outbreak. The exposed population was informed by the local newspaper and press releases on the municipality website to participate in the study. All inhabitants of the Vuorela and Toivala area were invited as well as those visiting or working in this area. Data was collected between 19^th^ July and 1^st^ August, 2012, and any person living or spending time during the study period in the defined outbreak area was eligible to participate in the study. Study participants were asked about their basic demographic characteristics, clinical symptoms (from 4^th^ to 20^th^ July, 2012) and habits of consumption of tap water in the defined outbreak area (from 1^st^ to 30^th^ July, 2012).

#### Data analysis

We used a commercial web based questionnaire from Webropol (www.webropol.fi). The data was analysed as univariate factors calculating the risk ratios for all risk factors asked and frequencies of the illness as a retrospective cohort sample using R [29]. Subsequently, a binary log and logistic regression models with case status as the outcome variable and those explanatory variables that were significant in the univariate analysis were included, the analysis were performed in R. Furthermore, as we had the information of all the inhabitants, we compared the age and sex between the non-cases and the source population using a standard Wald's statistic for calculating the confidence intervals for the observed difference in percentages between the groups.

#### Spatial analysis and regression

**Figure 1:**
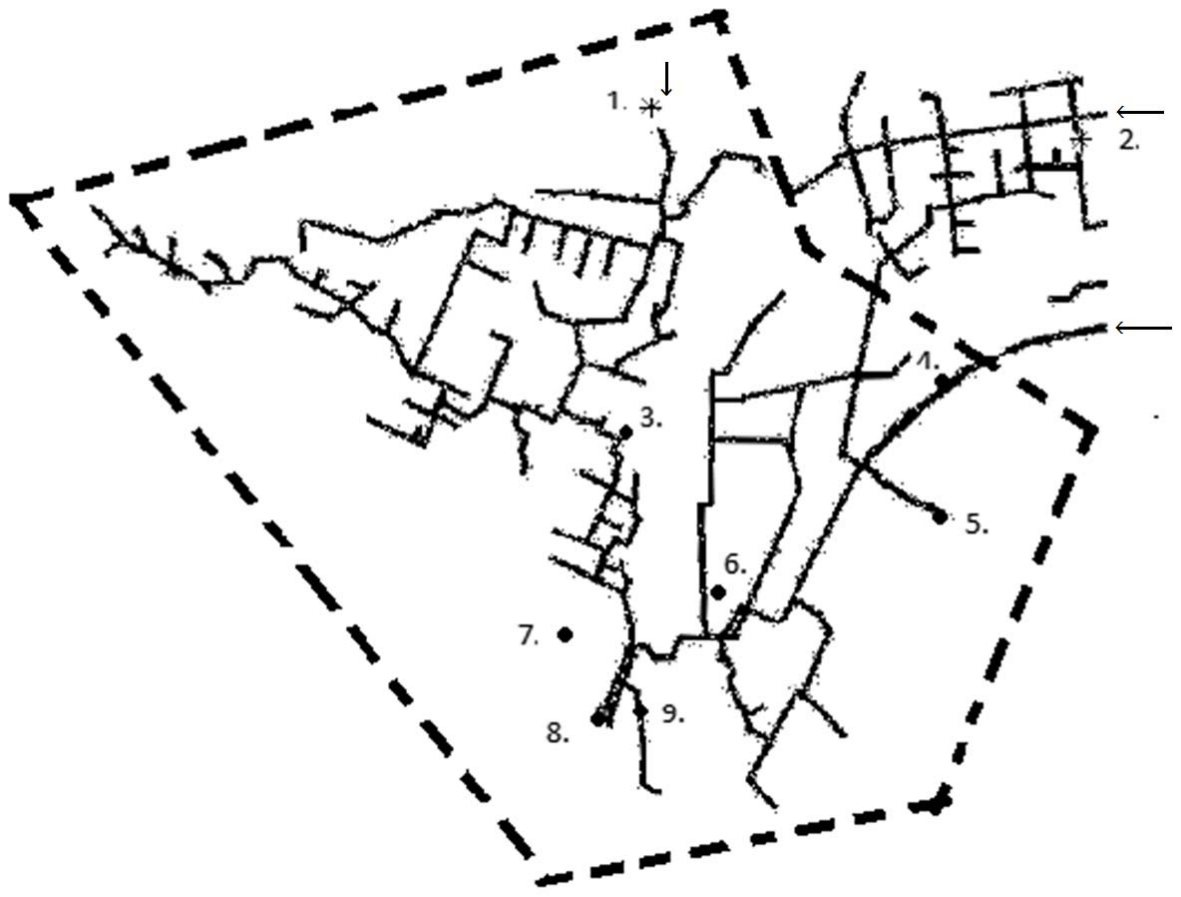
Schematic map of the water pipe of a defined outbreak area in Vuorela, July 2012. The outbreak (boil water notice) area is indicated by dashed line (- - -), the water sampling points (1–9) are coded as (•) with a positive culture finding and (*) with a negative finding. Arrows indicate the inflow points of the water from the water plant (outside the figure). Points 1,2,3,6,7 and 9 are tap water sampling locations, point 4 is the water pipe line breakage point (surface water), point 5 represent drinking water from the upper water storage reservoir and point 8 is the municipal effluent sampling location.

For the spatial analysis, only those persons with address information available from the National Population Register and who replied to the cohort study were included. We obtained information on name, address, date of birth and living location co-ordinates. Sampling locations in the water distribution pipeline were plotted on a schematic map obtained from the local construction office (Figure 1). The shortest distance via the water pipe to the pipe breakage was determined from the obtained digital map using an R package gdistance. The distance data was subsequently allocated to each person living in the area. We also included a spatial correlation variable for the model to explain the possible transmission of the infection within households and closely living contacts and due to the other possibly unmeasured spatially correlated factors. We evaluated the model by residual diagnostics, residuals were plotted against the predicted values and Cook's diagnostic values were calculated [30]. We subsequently categorised the distance to four equal groups and compared the proportion of ill persons in each.The statistical calculations were done in R using both routine and special spatial packages [29]. The code for calculating the spatial distance via the water pipe in R which is presented in (File S1).

### Clinical microbiology investigation

#### Microbiological analysis of patient samples

A total of 25 patients were sampled between 17^th^ July and 2^nd^ August, 2012. The age of the sampled patients had a median of 43 years (range 5–85 years). The potential causative agents were tested broadly, each from 5–21 samples aiming of qualitative detection of possible pathogens (Table 1). The initial samples were analysed in the local clinical laboratory with routine tests for common pathogens. As no major pathogens were detected, more samples were collected and analysed in national reference or specialized laboratories. A part of the patient samples were tested for enteric bacterial pathogens using standard clinical microbiology culture methods and PCR for the target genes of *Campylobacter jejuni/coli* (*rimR, gyrB*), *Salmonella* spp. (*invA*), *Shigella* spp./enteroinvasive *E. coli* (*ipaH, invE*) and *Yersinia* spp. (*virF, rumB*) [31]. The presence of diarrheagenic *E. coli* (i.e. EHEC, EPEC, ETEC, EIEC/*Shigella* spp. and EAEC) virulence genes (*pic*, *bfpB*, *invE*, *hlyA*, *elt*, *ent*, *escV*, *eae*, *ipaH*, *aggR*, *stx_1_*, *stx_2_*, *estlb*, *estla* and *astA*) were determined using multiplex PCR and qPCR techniques from mixed preliminary cultures grown on CLED (cystine lactose electrolyte deficient) agar medium plates [31], [32]. The *ipaH* and *invG* genes are specific for both *Shigella* spp. and EIEC and the tests do not distinguish between these two organisms. If PCR for EHEC genes was positive, the specific colony was picked out when possible from the mixed culture plate for a single PCR of the *stx* genes. Electron microscopy was performed to detect enteric viruses, such as noro-, rota-, adeno-, entero-, sapo- and astroviruses. In addition, for norovirus reverse transcription (RT)-qPCR [33], [34] was performed. For sapovirus analysis, two different PCR protocols were used [35], [36] and nucleic acid sequences were determined from the amplicons of the polymerase region [37]. Samples were also tested for enteric parasites *Giardia* and *Cryptosporidium* using enzyme immunoassay method for antigen detection [38]. Seven frozen faecal samples were tested in a retrospective testing scenario for the presence of *Arcobacter* spp. by culture method as described previously [39] and the plates were inspected regularly for up to 3 weeks [40]. A species-specific multiplex-PCR was performed for detection of *A. buzleri*, *A. cryaerophilus* and *A. skirrowii* as described previously [41]. The samples were also tested using an additional *Arcobacter* genus specific PCR method [42].

**Table 1:** Microbiological results of faecal samples from symptomatic patients of a waterborne outbreak in Vuorela, July 2012.

Microbial pathogen and methods used	Number of patients tested positive/number of tested
*Campylobacter jejuni/coli* 1 ^,^ 2	1^5^/21
*Salmonella* spp.^1^^,^ 2	0/21
*Shigella* spp.^1^^,^ 2	0/21
*Yersinia* spp.^1^^,^ 2	0/21
*E. coli*	
- EHEC^1^^,^ 2	2^6^/12
- EPEC^2^	6/12
- ETEC^2^	0/12
- EIEC/*Shigella* 1 ^,^ 2	0/12
- EAEC^2^	2/12
*Arcobacter* spp.^1^^,^ 2	0/7
Norovirus^2^^,^ 3	0^2^/12, 0^3^/17
Adenovirus^3^	0/17
Enterovirus^3^	1/17
Sapovirus^2^^,^ 3	5^2^^,^ 7/12, 3^3^/17
Astrovirus^2^^,^ 3	0^2^/7, 0^3^/17
*Giardia* 4	0/19
*Cryptosporidium* 4	0/19

Methods used were cultivation^1^, PCR/RT-PCR^2^ (polymerase chain reaction/reverse transcripition-PCR), EM^3^ (electron microscopy) and/or EIA^4^ (Enzyme Immunoassay). ^5^ *Campylobacter jejuni* and ^6^EHEC O157:H7 (from one sample) and ^7^sapovirus GII.P3 were isolated from the samples.

### Environmental investigation

#### Environmental sampling and analysis

The drinking water in the distribution network of Vuorela and Toivala area is UV-disinfected groundwater produced in the nearby Jäläniemi waterworks. The Siilinjärvi municipality owns and operates this public drinking waterworks and its distribution network. The employees of the Siilinjärvi municipality, including health protection authorities and personnel of the waterworks were responsible of environmental sampling and delivery of samples to expert laboratories. The water quality management routines include testing for coliform bacteria, heterotrophic plate counts and *Clostridium perfringens* at the waterworks and around the water distribution network according to European regulation [43]. The water quality results were acceptable prior to the outbreak with the last results dating back to 7^th^ June and 2^nd^ July, 2012. After accidental pipe breakage at 4^th^ July, the pipeline was fixed within 14 hours, then flushed and sampled from two points (locations before the breakage point outside the schematic map and after the breakage point, point 6 in Figure 1) in 5^th^ July, 2012 for indicator bacteria testing. The water quality was shown to comply the legal microbiological quality criteria (i.e. absence of coliform bacteria, *E. coli* and intestinal enterococci with no abnormal change in heterotrophic plate counts, Table 2).

**Table 2:** The counts of water quality indicator bacteria (range of MPN or CFU/100 mL), occurrence of faecal pathogens and chlorine concentrations in the water samples taken in July and August, 2012 in Vuorela, Finland.

Date (number of samples)	*E. coli*	Coliform bacteria	Entero-coccus	Faecal pathogens	Chlorine (mg/l)
*Drinking water distribution system samples from the clean area*					
5–19 Jul (12)	0	0–1	0	Not detected/3–6 samples^1^	ND, 0.4–1.8^2^
*Drinking water distribution system samples from the boil water notice area*					
5 Jul (1)	0	0	ND	ND	ND
16 Jul (4)	0–150	0–150	0–17	*Arcobacter* spp./1–4 samples^3^	ND
17 Jul (4)	0–21	0–34	0–2	EHEC, EPEC and EAEC virulence genes^4^	ND
18–20 Jul (10)	0^5^	0	0	Not detected/2–4 samples^1^	<0.1–1.6
23–30 Jul (13)	ND^6^	ND	ND	Not detected/3 samples^7^	<0.1–2.0
1–29 Aug (9)	ND^6^	ND	ND	ND	0.4–1.9
*Water storage reservoir*					
17 July (1)	110	190	15	Norovirus and adenovirus^8^	ND
21 July (1)	0	0	ND	Not detected^9^	ND
*Biofilms from the water meters removed from the boil water notice area*					
1 Aug (2)	ND	ND	ND	*Arcobacter* spp.^10^	ND
*Raw water at the groundwater abstraction plant*					
9 August (1)	0	0	ND	Not detected^11^	ND
*Surface water samples (contaminant source)*					
23 Jul (1)	86	450	44	*Campylobacter jejuni* 10–100 cfu/l, EPEC virulence genes^12^	ND
29 Aug (5)	0–94	ND	5–80	ND	ND
*Community wastewater*					
25 Jul (1)	ND	ND	ND	Sapovirus	ND

ND; not determined.

1 A portion of the samples were selected for *Salmonella*, *Campylobacter*, enterohaemorragic *E. coli* (EHEC) culture analyses and for norovirus analysis.

2 Measured from three locations at 19 July.

3 Samples were tested for noro-, adeno-, rota- and sapoviruses, *Campylobacter* and *E. coli* virulence genes. *Arcobacter* was tested from DNA extracts and genus specific PCR was positive in one sample (point 9 in Figure 1).

4 *E. coli* virulence genes were detected after ultrafiltration from one sampling location (point 5 in Figure 1). *Salmonella*, *Campylobacter*, EHEC (culture method), noro-, rota- and sapovirus, *Giardia* and *Cryptosporidium* were not detected (1–4 samples tested/method).

5 One colony of *Clostridium perfringens* was found from 1 000 mL of tap water sample taken from the most contaminated area.

6 *Clostridium perfringens* was analyzed and not detected from 5 000 mL samples.

7 Samples were tested for sapovirus.

8 Sample was tested for noro-, adeno-, rota- and sapoviruses, *Salmonella*, *Campylobacter* and *E. coli* virulence genes. *Clostridium perfringens* was detected (10 CFU/L).

9 Sample was tested for *Campylobacter* and noroviruses. *Clostridium perfringens* was detected (2 CFU/L).

10 Sample tested for *Campylobacter*, *Arcobacter*, *Giardia* and *Cryptosporidium*.

11 Sample tested for *Campylobacter*.

12 Sample tested for *Campylobacter*, *E. coli* virulence genes, noro- and adenoviruses, *Giardia* and *Cryptosporidium*. *Clostridium perfringens* was detected (40 CFU/L).

A total of 65 water samples were collected between 5^th^ July and 29^th^ August (Figure 1). The majority of samples (n = 54) were tap water samples collected from various parts of the distribution system (1–10 sampling events/location) (Table 2). Also a raw water sample from a water production well, two biofilm samples from water meters and a sample from the community wastewater influent were collected. Surface water from a rainwater collection well and ditches at the pipeline breakage location (n = 5) were sampled as potential contamination sources. Both small (500–2000 mL) and large (10 L) scale water samples were analysed for pathogens. On-site large volume sampling (more than 100 L) using tangential flow ultrafiltration was conducted 17^th^ July at one of the distribution system sites where high counts of faecal bacteria were detected at the previous day small scale sampling (point 7 represented in Figure 1). The ultrafiltration was conducted without sodium polyphosphate using a semi-automated system [17], [18] and the bovine-serum pre-treated ultrafilter was eluted after sampling with 9∶1 retentate/elution solution and 500 ml elution solution [17], [44].

Culture methods were used to analyse *E. coli* and coliform bacteria [45], [46], intestinal enterococci and heterotrophic plate counts [46], and vegetative cells and spores of *Clostridium perfringens* [47] from water samples. The presence/absence of faecal bacterial pathogens, thermotolerant *Campylobacter* spp. and *Salmonella* spp., was determined using culture-based selective enrichment methods [46], [48], and EHEC was analysed using serotype O157 specific immunomagnetic separation coupled with the selective culture enrichment [49]. Enteric viruses were concentrated as previously described [50], except using Sartolon polyamide membranes (diameter 47 mm, pore size 45 µm; Sartorius, Goettingen, Germany). Norovirus, rotavirus and adenovirus were analysed using previously described RT-qPCR and qPCR methods [48], [50], [51]. For sapovirus analysis, the faecal protocol was applied to water as described earlier. Protozoan parasites *Giardia* and *Cryptosporidium* were determined from a tap water concentrate using immunomagnetic separation and epifluorescence microscopy according to the international standard method [52] and from water meter biofilm samples using molecular techniques [53]. The concentration of free and total chlorine in the drinking water distribution was measured on-site using the Palintest Micro 1000 photometer and in the laboratory using ISO 7393 standard method [54].

#### Further identification of pathogens

*E. coli* isolates from seven tap water samples (n = 19) and DNA extracts (n = 4) were screened for presence of virulence genes of EHEC, EPEC, ETEC, EIEC and EAEC [31], [32]. In addition, the same virulence genes were tested from mixed cultures (tap water and rainwater sample) collected from membranes during the *E. coli*/coliform analysis. PFGE-profiles of *C. jejuni* isolates (n = 6) from patient and environmental samples were produced using *Kpn*I and *Sma*I restriction enzymes [55].

Extracted nucleic acids from total (DNA) and active bacterial fraction (RNA) in water samples were used as template to amplify faecal source identifiers and the bacterial 16S rRNA gene for microbial source tracking (MST) and NGS applications, respectively. MST assays were performed as previously described [21]. The samples were obtained from the upper storage reservoir water before and after cleaning (point 5 in Figure 1) and from a tap water sample collected during the contamination episode (point 7 in Figure 1). The MST assays included the analysis of faecal bacterial groups *Bacteroidales* spp. (GenBac3 assay [56]) and human-specific *Bacteroidales* (HF183 assay [57]).

For Illumina MiSeq NGS, we utilized barcoded primers 515F and 806R to produce 250 bp pair-ended sequences [58]. Reads were processed and analyzed using the software MOTHUR v1.30.2 ([59]; http://www.mothur.org) as described previously [60]. Briefly, fastq files with forward and reverse reads were used to form contigs. The reads that met the following criteria were excluded from further analysis: the length was no greater than 255 bp; contained ambiguous bases (N) or homopolymers greater than 7 bases; identified as chimera; or classified as Chloroplasts or Mitochondria. Reads were aligned and sorted with >97% similarity into operational taxonomic units (OTUs). Taxonomic classification was obtained using the tool Classifier in the Ribosomal Database Project II release 10.28 [61]. The raw reads were deposited in the NCBI Sequence Read Archive (SRA) under accession number SRP041117. Using MEGA v5.2 [62], a phylogenetic tree based on the aligned 16S rRNA gene sequences (∼255 bp) was constructed with the Maximum Likelihood (ML) method using Tamura-Nei model [63] with 1,000 bootstrap replicates. The tree was used to infer the phylogenetic relationship among sequences classified as *Arcobacter* obtained in this study. *Sulfurospirillum deleyianum* (NR_074378) and *Campylobacter fetus* (L04314) were used as outgroup. Three water samples and two water meter samples were subsequently analysed for *Arcobacter* spp. using genus specific PCR [42] and species specific multiplex-PCR [41] from the DNA extracted as previously described [50].

## Results

### Epidemiological investigation

#### Descriptive analysis

**Figure 2:**
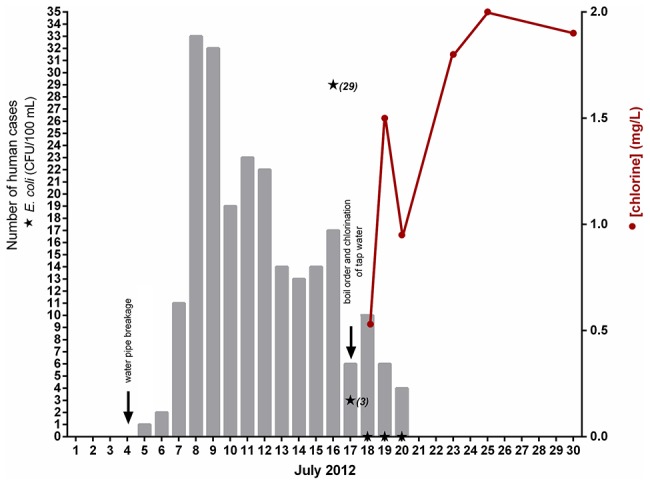
Epidemic curve of a waterborne outbreak in Vuorela, July 2012 based on the reported onset date of illness of the cases, and *E. coli* bacteria counts and chlorine levels in the point 7 (See Fig. 1) of the water distribution network.

Of all 2931 inhabitants (source population) of the defined outbreak area, 473 (16%) persons participated in the study (study population). We excluded 19 persons absent from the outbreak area during the whole study period and 23 due to travelling abroad. In total, we identified 225 cases and 206 healthy persons from the cohort study according to the case definition. However, for the individual risk factors data contained a few missing values. During the four week period after the water distribution breakage, an estimated 800 excess persons visited either a nurse or GP in the local health care centre. The outbreak curve presented in Figure 2 implicates a point source outbreak.

#### Demographic characteristics

Of the 431 respondents, 33% (135/408) were male. The mean age was 41.2 years (range 1–80 years). As there were few children among the respondent (10 in total, 3 among non-cases), we excluded the children from comparison analysis. The proportion of male (33%, 60/182) among the non-cases in the study population was lower to that of the source population (48%, 1074/2252). This difference for the male proportions of 14.5% (7.4%–21.6%) was statistically significant. Persons were slightly older among the source population compared to the study population, Table 3. Also, there were 50.2% (232/462) households (unique water delivery points) with at least one person under 18 years of age in the source population compared to 68.5% (126/184) among the study population. This difference was significant, CI for the difference was 18.3% (10.2%–26.4%). Therefore, households with children were more likely to respond to the study.

**Table 3:** Comparison of the study population to source population with respect to age groups in a waterborne outbreak in Vuorela, July 2012.

Age group	Study population (non-cases)% of population (number of persons)	Source population % of population (number of persons)	% difference (95% confidence intervals)
20–39 years	40% (78/195)	29% (655/2252)	11% (3.9%–18.1%)
≥40 years	60% (117/195)	71% (1597/2252)	−11% (−18.1%–−3.9%)

The course of the illness was mild, only one person was admitted to hospital. The main symptoms were stomach ache 88% (199/225), nausea 85% (191/225) and diarrhoea 82% (185/225). The length of the illness had a median of 3 days (range 1–30), 16% (35/225) of the cases sought for medical assistance and one patient was hospitalized (37-year old female with no underlying medical condition). Absence from work due to outbreak illness was reported by 31% (133/431) of the study participants, the total number of working days lost because of illness was 398. Taking care of a sick child was not included.

#### Univariate analysis of the cohort

Of all possible cases occurring in the area, 225 cases responded to the study in an area with a population of 2931 persons. Of the water related risk factors, drinking untreated tap water in the outbreak area had a risk ratio of 5.6 (95% CI 1.9–16.4), also drinking untreated tap water at home RR 2.2 (95% CI 1.2–4.1) and outside home RR 1.6 (95% CI 1.2–2.0) in the outbreak area were associated with illness, Table 4. Drinking boiled water at home was a protective factor, RR 0.69 (95% CI 0.53–0.90), drinking well water or bottled water were not significant either at home or outside home. It was observed that the risk ratio increased as the number of glasses of water consumed increased. This further strengthened the role of the contaminated tap water as a cause of the outbreak in a dose response manner, table 5.

**Table 4:** The Univariate and multivariate results for individual risk factors and the generalized additive model risk ratios with the spatial term of a waterborne outbreak in Vuorela, July 2012.

	Explanatory variable	Risk ratio or univariate log regression exp (β-values), (95% confidence intervals) for individual risk factors	Multivariable generalized additive logit model, exp (β-values), (p-value)
Personal characteristic	Age (continuous in years)	0.99 (0.98–0.99)	0.975 (0.0061)
Drinking at home	Tap water	2.2 (1.2–4.1)	5.90 (0.0037)
	Water from own well	1.0 (0.57–1.87)	n/a
	Bottled water	0.86 (0.69–1.07)	n/a
	Boiled water	0.69 (0.53–0.90)	n/a
Drinking water in Vuorela (outside home)	Tap water	1.6 (1.2–2.0)	n/a
	Water from own well	0.83 (0.56–1.22)	n/a
	Bottled water	0.90 (0.72–1.12)	n/a
	Boiled water	0.80 (0.56–1.15)	n/a
Spatial variables	Distance from the breakage by waterpipe (metres)	0.99950 (0.99930–0.99969)	0.998 (0.060)
	Spatial variable (coordinates)	n/a	n/a (0.002)

**Table 5:** The dose-response between the illness and the amount of water consumed at home in a waterborne outbreak in Vuorela, July 2012.

Number of glasses of water consumed at home per day	Cases/total number of persons in the implicated group (%)	Risk ratio (95% confidence intervals)	p-value
0	6/28	reference	
1–3	49/114 (43%)	2.01 (1.06–4.81)	0.065
4–6	112/193 (58%)	2.71 (1.48–6.40)	0.0066
7–9	37/48 (77%)	3.60 (1.94–8.54)	0.00054
10 or more	9/19 (47%)	2.21 (0.96–5.68)	0.068

Those not drinking water at home served as a control group.

#### Multivariate and spatial analysis of the cohort

Of those fulfilling eligibility criteria, 20 participants had no address information, 81 lived outside the defined boil water notice area and 17 could not be found in the national population register, leaving 313 persons for the spatial analysis. After these exclusions, 154 cases and 159 non-cases were used for the spatial analysis. Age, drinking tap water at home and distance from the breakage point were significant in the multivariable model, Table 4. Younger persons were more likely to become ill (p = 0.0061), risk also increased if tap water was consumed at home (p = 0.0037). Distance from the leakage point was inversely associated with becoming a case (p = 0.060). Also spatial variable was significant in the generalized additive model (p = 0.002), this is likely to reflect the person to person transmission within households or neighbourhoods (Table 4). The distance via the water pipe was shorter for the cases compared with the non-cases (Table 4). This indicates that the closer one lived to the water breakage point, the more contaminated the drinking water was and therefore the likelihood of the illness was higher. The diagnostics of the model for randomized quantile residuals were normally distributed to suggest correct specification of the model. Between the categorised groups, the proportion of ill persons was higher among those living closer to water breakage point, Table 6.

**Table 6:** Categorized distance and proportion of cases within those groups in a waterborne outbreak in Vuorela, July 2012.

Distance categories	% (Cases/total)
Distance 1 (<2332)	72.4% (63/87)
Distance 2 (2332–2713)	45.5% (35/77)
Distance 3 (2713–3202)	43.5% (37/85)
Distance 4 (>3202)	29.7% (19/64)

### Clinical microbiology investigation

#### Patient faecal samples

The microbiological analysis of faecal samples from the patients identified several pathogens (Table 1). The pathogens identified included sapovirus (detected from 5 patients of 12 tested, 2 sapoviruses were genotyped as GII.P3), enterovirus (detected from one patient out of 17 tested) and *C. jejuni* (isolated from one patient out of 21 tested). Two sapovirus nucleic acid sequences were submitted to Genbank with accession numbers KJ200380 and KJ200381. In addition, specific virulence genes of pathogenic *E. coli* were detected, including genes from EHEC (2/12), EPEC (6/12) and EAEC (2/12). In subsequent culturing of the virulence gene positive samples, one *E. coli* O157:H7 positive culture was found. No suspect *Arcobacter* spp. were detected by culture methods or by PCR.

### Environmental investigation

#### Faecal microbes in the drinking water distribution

Faecal indicator bacteria were detected in four out of 11 tap water samples taken from different sampling points of the distribution on 16th and 17th July, 2012 (Table 2). The bacterial counts from the drinking water reservoir sample (point 5 in Figure 1) taken at the same time were high for total coliform bacteria, *E. coli* and enterococci. In addition, low numbers of adenoviruses and noroviruses (genogroup II) were detected in the storage reservoir sample. Water samples tested negative for sapovirus, although the community waste water influent was shown to contain sapovirus. Specific virulence genes of EHEC, EPEC and EAEC were detected from the large volume tap water sample (point 7 in Figure 1).

#### Cause of the water contamination

Technical investigations revealed that the water storage reservoir had been rapidly filled with contaminated water after the pipe breakage repair. The microbial contaminants unintentionally funnelled to the storage reservoir could remain viable as no disinfection was used in the distribution system (Table 2). Subsequently the contaminated water was introduced to the Vuorela and Toivala distribution system due to the water usage. The water level in the storage reservoir varies depending on water usage and pumping of the fresh water from the groundwater abstraction plant into the reservoir. The water quality at the groundwater abstraction plant was tested acceptable and free of microbial contaminants shortly after the breakage (Table 2). De-contamination of the reservoir was ensured by emptying and then mechanical cleaning, washing and chlorination of the inner surfaces of the storage reservoir. Indicator bacteria were no longer detected from the water distribution system after flushing and successful chlorination (Table 2). However, it took approximately a week before *C. perfringens* spores were absent in the water samples from the contaminated distribution system and chlorine levels reached the minimum target concentration (0.5 mg/l) at the most distant parts of the distribution.

#### Microbial source tracking

GenBac3 and HF183 assays showed the presence of *Bacteroides* spp. and human-specific *Bacteroidales*, respectively, in the contaminated tap water and in the storage reservoir (Table S1). There were more rRNA markers present compared to the rRNA gene (DNA) markers (rRNA∶rDNA ratios were higher) in the storage reservoir sample than in the contaminated tap water sample. After cleaning of the storage reservoir, the GenBac3 and HF183 marker concentrations declined to below the quantification and detection limits, respectively (Table S1). A reactive surface water sample from the rainwater well was collected one week after the outbreak notification to evaluate the potential environmental sources near the pipe breakage location (Table 2). *C. jejuni* and EPEC virulence genes were identified from the surface water sample. Also indicator bacteria; coliforms, *E. coli* and intestinal enterococci were abundant. Five *C. jejuni* isolates from this suspected contamination source had identical *Kpn*I and *Sma*I profiles in PFGE, but different from those of the *C. jejuni* patient isolate (data not shown).

#### Bacterial communities in water

**Figure 3:**
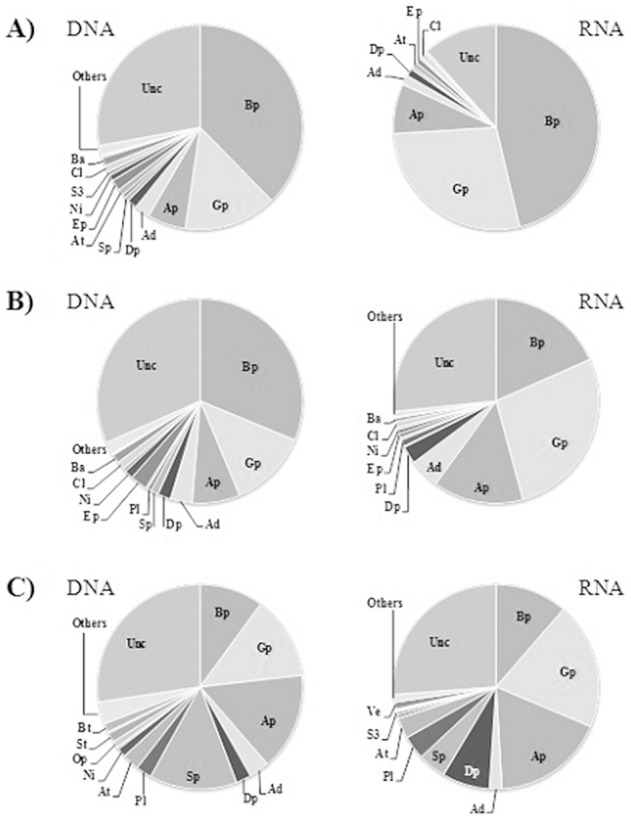
Distribution of the Bacteria domain as determined by taxonomic identification of partial 16S rRNA gene sequencing (at class level) in a waterborne outbreak in Vuorela, July 2012. Charts and tables represent the cumulative distribution of total DNA and RNA extracted from samples of A) the upper storage reservoir before cleaning (point 5 in Figure 1), B) tap water during contamination (point 7 in Figure 1) and C) the upper storage reservoir after cleaning. Legend: Beta-proteobacteria (Bp); Gamma-proteobacteria (Gp); Alpha-proteobacteria (Ap); Acidobacteria (Ad); Delta-proteobacteria (Dp); Sphingobacteria (Sp); Planctomycetacia (Pl); Actinobacteria (At); Epsilon-proteobacteria (Ep); Nitrospira (Ni); Verrucomicrobia Subdivision3 (S3); Clostridia (Cl); Bacteroidia (Ba); Opitutae (Op); Spartobacteria (St); Verrucomicrobiae (Ve); Bacteroidetes (Bt); Others (classes each representing <1%); unclassified (Unc).

Community analysis based on the bacterial 16S rRNA region showed a higher diversity for contaminated sampling sites, while upper storage reservoir after the cleaning showed a lower diversity (Table S2). It is noteworthy that the community diversity measured as a total number of OTUs in the community was higher within the DNA reads than within the RNA reads (Table S2). Taxonomic analysis of contaminated water samples indicated a high presence of the class Beta-proteobacteria including the family *Comamonadaceae* and genus *Zoogloea*, and Gamma-proteobacteria including affiliated family *Methylococcaceae* and genera *Methylobacter* and *Pseudomonas* (Figure 3, A and B; Table S3).

**Figure 4:**
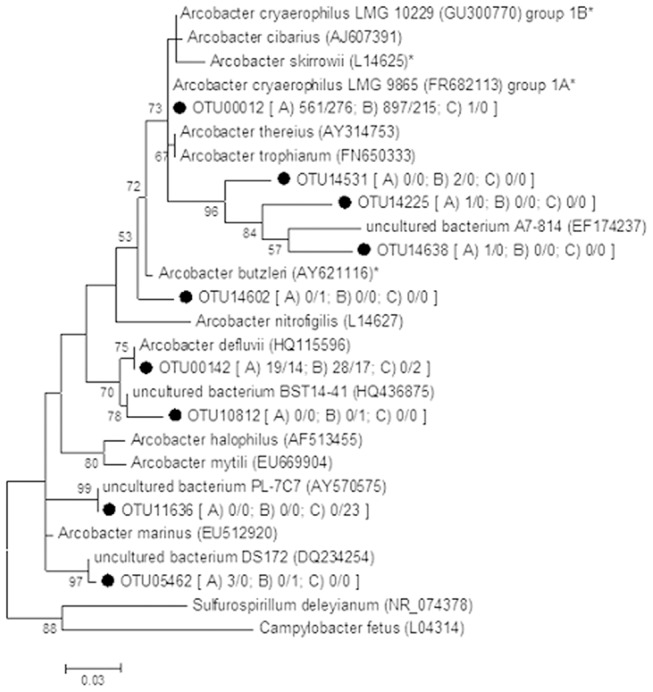
Phylogenetic relationships among OTUs (•) of the genus *Arcobacter* in a waterborne outbreak in Vuorela, July 2012. The tree was inferred from a maximum likelihood analysis of aligned 16S rRNA gene sequence (≈255 bp) and nodes with a bootstrap value ≥50% of 1 000 replicates are identified. *Sulfurospirillum deleyianum* (NR_074378) and *Campylobacter fetus* (L04314) were used as outgroup. Number in bracket represents the total amount of DNA/RNA reads identified in samples from A) the upper storage reservoir before cleaning, B) tap water during contamination and C) the upper storage reservoir after cleaning. *A. butzleri, *A. cryaerophilus* and *A. skirrowii* have been associated with gastrointestinal diseases [76].

Interestingly, also a significant share of reads generated from the contaminated water samples affiliated with Epsilon-proteobacteria genus *Arcobacter* (Table S3 and Table S4). Further sequence analysis revealed close relationship to *A. cryaerophilus* group 1A LMG9865 (OTU00012 in Figure 4). However, the corresponding tap water sample was negative for the species-specific *Arcobacter* multiplex-PCR (for *A. butzleri*, *A. cryaerophilus*, *A. skirrowii*) and the storage reservoir samples were no longer available for species-specific testing. *Arcobacter* genus specific PCR was positive for a tap water sample and a water meter sample collected from another location (point 9 in Figure 1) but these samples remained negative by culture. Although the sequences from both samples (Genbank with accession number KJ196910) showed high sequence similarity (>98%) with *A. cryaerophilus* (uncultured), *A. skirrowii* and *A. cibarius*, no species attribution can be made.

After the cleaning and chlorination of the upper storage reservoir, the relative abundance of Beta- and Gamma-proteobacteria decreased and the abundance of *Sphingobacteria* including genus *Pedobacter* increased (Figure 3, C; Table S3). The reads associated *Bacteroides*, *Escherichia* and *Clostridia* in the contaminated water samples were absent from the storage reservoir sample after cleaning and chlorination (Table S4).

## Discussion

This study describes a large-scale municipality area wide outbreak due to breakage of drinking water distribution pipeline during a road construction work. The illness in the community became apparent only two weeks after the incident but the cause of the illness was obvious. This was confirmed using a polyphasic approach applying microbiological, epidemiological and statistical methods. Multiple causative microorganisms were isolated from patient samples. No major definite causative pathogen was identified but sapovirus was most frequently detected in patient samples. Also various types of virulent *E. coli*, *C. jejuni* and *Arcobacter* spp. might have played a role in the onset of gastrointestinal symptoms. All these organisms have been previously associated with waterborne transmission [5], [9], [12], [13], [23]. By applying the spatial and microbiological analysis, we could identify the contamination route for this outbreak. The distance to the water breakage point was inversely associated with illness both by epidemiological and spatial methods. We further confirmed the role of the contaminated water as a vehicle by novel spatial analysis. The spatial method provided here could be used to compare the likelihoods between possible candidate point source locations. Effective control measures and rapid and continuous communication with the media were essential to ensure appropriate public health measures.

The overall clinical illness in most patients was mild with only 16% accessing medical assistance and only one patient being hospitalized. Majority of the cases had gastrointestinal symptoms with mild course and relatively short length. Overall, the number of cases was likely to be an underestimate for the whole population. The case definition used in the present study was relatively sensitive due to the mild overall nature of the illness. The contaminated water consumed whether at or outside home in the defined outbreak area was associated with illness. We further showed by epidemiological methods that drinking contaminated tap water was associated with illness in a dose-response manner. The effect levelled off at the highest dose, often observed in food or waterborne outbreaks [64], [65], this has been observed also in previous studies [1], [3], [9]. Furthermore, drinking boiled water was a protective factor indicating the effectiveness of the implemented control measures.

It is challenging to estimate the true attack rate in the present outbreak. It is quite likely that people showing symptoms responded more actively in the study compared to non-cases. We had a unique opportunity to compare the study population to the source population and found that women responded more actively to the study. This has been found also previously [66]–[68]. As we also found slightly more younger people among the non-cases compared to the source population, therefore, the observed effect of age should be interpreted with caution. The crude attack rate obtained from the questionnaire study (225/431, 52%) is within the same range to what has been observed in similar outbreaks, namely between 31–88% [2]–[4]. The true number of ill persons is difficult to estimate, but the educated evaluation of the local general practitioner (800 cases) is a fair estimate.

By applying the spatial and microbiological analysis, we could identify the contamination route for this outbreak. The contaminated water from the breakage initially filled the water storage reservoir and subsequently this water was distributed to the community over a number of days. The drinking water storage reservoirs have contributed to the transmission of waterborne infections also elsewhere, usually due to the improper maintenance and structure of the reservoirs [46], [69], [70]. In our case, the exact cause of the contamination was pipeline breakage at the road construction site. The storage reservoir prolonged the contaminant transport time to the water consumers. The likelihood of illness was higher closer to the water breakage point as measured by water pipeline length. Also the higher amount of water consumed in the boil water notice area increased the likelihood of illness. Additionally, younger persons (i.e. parents of small children) might have been more likely to become infected presumably due to the fact that they had more contact to young infants.

The source of the illness was obvious from the beginning of the outbreak, but we initially searched for one major pathogen as a causative agent yet aware that this type of outbreak is often caused by multiple pathogens. The sapovirus finding was novel and has rarely been detected in previous Finnish waterborne outbreaks [13]. According to recent reports, sapoviruses can be commonly found in environmental waters [10], [71], [72] highlighting the importance of including sapovirus detection into the outbreak investigations. Sapoviruses cause acute gastroenteritis primarily among young children [36], and indeed, young age was suspected to be one risk factor in this study. This result, together with the presence of sapoviruses in communal wastewater influent, support the assumption that sapovirus was commonly circulating in the community. We did not detect sapoviruses in the drinking water samples, presumably due to delayed sampling and low viral concentration in the drinking water. We also found that adults had symptomatic sapovirus infections. Adults have been found to have sapovirus infections in other studies [12], [73], [74]. The occurrence of human infecting sapoviruses among the patient cases together with the detection of human-specific genetic marker HF183, adenoviruses and noroviruses from the contaminated drinking water suggests that human faecal material was present at the pipe breakage site. No leakage was identified in a waste-water pipe line located in the same construction pit as the drinking water pipeline. Instead, it was concluded that surface water runoff from the nearby recreational area was the potential source of the faecal microbes. The abundant pathogenic *E. coli* findings from patient and water samples were not surprising bearing in mind the nature of the contamination. One positive *C. jejuni* finding in a patient may have had sporadic origin as the environmental isolate was of different type.

We used a novel NGS approach to study the microbial communities in the tap water distributed in the contaminated water pipe network. To our knowledge, this was the first time that high-throughput sequencing was employed for detection of potential causative bacterial agents in a waterborne outbreak case. By using the novel microbial community analysis for the water samples, we could show an abundance of *Arcobacter* spp. in the drinking water distribution during the contamination. Further analysis found high sequence similarity with potential pathogenic *Arcobacter* spp. (*A. cryaerophilus*, *A. skirrowiii* and *A. cibarius*). Although the abundance of reads of *Arcobacter* correlated with the quality of water (increase in contaminated waters), the resolution of *Arcobacter* species lineage was not possible due to ambigious nature of the 16S rRNA sequences [75]. Since *Arcobacter* spp. have been suspected as a cause of gastroenteritis in humans [5], [76], we aimed to isolate these from the remaining patient samples. No *Arcobacter* spp. were isolated perhaps due to the small quantity, long storage time (more than one year) or storage conditions of the samples. Nevertheless, considering that there is no gold standard for the isolation of these putative pathogens, their presence cannot be ruled out.

The NGS approach also provided information on the bacterial communities in the non-chlorinated and faecally contaminated drinking water. Until now, the analysis of indigeneous microbial community composition in non-chlorinated drinking water distribution systems has been scarce [77]. The detected change in the storage reservoir bacterial community structure after the cleaning and chlorination proves the effectiveness of these measures. Moreover, our result showing higher community diversity within the DNA reads than within the RNA reads suggest that DNA-based methods may not effectively discriminate between active and dormant populations. However, more work is needed to better characterize the bacterial community changes in the different drinking water distribution systems. For example, understanding the role of drinking water retention time in the network [77] and the effect of drinking water treatment processes such as filtration on the bacterial communities in the distribution [78] would facilitate the sustainable management of the microbial water quality in the distribution networks.

We further confirmed the role of the contaminated water as a vehicle by novel spatial analysis. By calculating the distance for each household to the water breakage point via the water pipe, we could show that the probability of illness decreased by the increasing length of the water pipe. However, it should be noted that non-symptomatic persons in the same households and neighbourhoods participated more actively in the cohort study. We used all cases in the model, including potential secondary cases. As the spatial term describes just secondary spread and was significant in the spatial logistic regression model, it suggests that there were indeed a number of secondary cases among the case patients. In addition to distance from the breakage point, age was inversely associated with illness and consumption of water in the Vuorela area was positively associated in a spatial logistic regression.

The study was limited by a slow response due to notification delay as it took a relatively long time before the outbreak was detected and confirmed. Therefore, the causative agents could not be isolated from the water samples. The microbial community analysis also warrants further studies. We used 16S rRNA as a target of the NGS studies. In the future, with careful design of pathogen specific primers for sequencing purposes, it might be possible to abandon the requirement of culture isolation for genotyping purposes. In successful NGS applications, the huge number of reads per sample potentially enables the identification phylogenetic relatedness between the causative pathogenic strains in different samples [79]. Subsequently, this might allow the use of NGS approach for source tracking and understanding of the transmission route of an outbreak.

## Conclusions

We used novel and existing statistical, microbiological and spatial methods to characterize a community wide waterborne outbreak. These methods may be applied to wide range of food and waterborne outbreak investigations and beyond. In particular, microbial community analysis in combination with traditional culture and PCR based methods aided to clarify the potential causative agents. In addition, the cohort study was carried out as a rapid web based application added with a novel spatial method to show the statistical association to the suspected point source leakage of the water pipe network. We also confirmed previous observations that women and younger people were more likely to respond to this type of study. In the present outbreak the event associated with the outbreak was fairly obvious, but in many outbreak situations the source cannot be easily identified [3]. Failures in the water distribution networks are common causes of waterborne outbreaks [2]. The method provided here could be used to compare the likelihoods between possible candidate point source locations.

## Supporting Information

Table S1 **Numbers of the GenBac3 and HF183 markers (log_10_ copies 100 mL^−1^) in TaqMan rRNA-targeted RT-qPCR (rRNA) and rRNA gene-targeted qPCR (rDNA) assays.**
(DOC)

Table S2 **Community diversity estimates (±CI) of the domain** ***Bacteria*** **.**
(DOC)

Table S3 **Taxonomic affiliation of the most abundant** ***Bacteria*** **domain representatives.**
(DOC)

Table S4 **Abundance of reads associated with faecal bacteria and/or disease agents detected in the Vuorela and Toivala drinking water distribution system.**
(DOC)

File S1 **R code for calculating the shortest direct distance and the distance via the water pipe between each inhabitant location and water pipe breakage point.**
(DOC)
