# Supplementary material for: Novel Microbiological and Spatial Statistical Methods to Improve Strength of Epidemiological Evidence in a Community-Wide Waterborne Outbreak
Source: PLoS One. 2014 Aug 22;9(8):e104713. doi: 10.1371/journal.pone.0104713 (PMC4141750; doi:10.1371/journal.pone.0104713)
Supplement: Table S1 — Numbers of the GenBac3 and HF183 markers (log10 copies 100 mL−1) in TaqMan rRNA-targeted RT-qPCR (rRNA) and rRNA gene-targeted qPCR (rDNA) assays. (DOC) [file pone.0104713.s001.doc]

Table S1. Numbers of the GenBac3 and HF183 markers (log_10_ copies 100 mL^-1^) in TaqMan rRNA-targeted RT-qPCR (rRNA) and rRNA gene-targeted qPCR (rDNA) assays.

| Target | The upper storage reservoir^1^ | Tap water during contamination^2^ | The upper storage reservoir after cleaning |
| --- | --- | --- | --- |
| General *Bacteroidales* (GenBac3) | | | |
| - rRNA | 4.75 | 4.11 | < LOQ |
| - rDNA | 3.96 | 4.00 | < LOQ |
| *rRNA:rDNA ratio* | *6.1* | *1.3* | *-* |
| Human-spesific *Bacteroidales* (HF183) | | | |
| - rRNA | 2.61 | 2.09 | < LOD |
| - rDNA | 2.63 | 2.69 | < LOD |
| *rRNA:rDNA ratio* | *1.0* | *0.3* | *-* |

^1^Sampling point 5, see Fig. 1. ^2^Sampling point 7, see Fig.1. LOQ, limit of quantification. LOD, limit of detection.
