# Supplementary material for: Novel Microbiological and Spatial Statistical Methods to Improve Strength of Epidemiological Evidence in a Community-Wide Waterborne Outbreak
Source: PLoS One. 2014 Aug 22;9(8):e104713. doi: 10.1371/journal.pone.0104713 (PMC4141750; doi:10.1371/journal.pone.0104713)
Supplement: Table S2 — Community diversity estimates (±CI) of the domain Bacteria . (DOC) [file pone.0104713.s002.doc]

Table S2. Community diversity estimates (±CI) of the domain *Bacteria*.

| Sample^1^ |  | Template | S^2^ |  | Richness estimators | |  | Diversity index | |
| --- | --- | --- | --- | --- | --- | --- | --- | --- | --- |
|  |  |  |  |  | ChaoI | S_ACE_ |  | Shannon (*H*) | Evenness (*E_H_*) |
| The upper storage before cleaning |  | DNA | 3 263 |  | 4 667 ± 207 | 4 719 ± 169 |  | 5.61 ± 0.03 | 0.693 |
|  |  | RNA | 2 334 |  | 5 302 ± 470 | 8 009 ± 393 |  | 4.23 ± 0.03 | 0.546 |
|  |  |  |  |  |  |  |  |  |  |
| Tap water during contamination |  | DNA | 3 873 |  | 6 011 ± 271 | 7 613 ± 259 |  | 5.95 ± 0.03 | 0.720 |
|  |  | RNA | 3 055 |  | 5 620 ± 360 | 7 735 ± 317 |  | 5.56 ± 0.02 | 0.692 |
|  |  |  |  |  |  |  |  |  |  |
| The upper storage after cleaning |  | DNA | 1 129 |  | 1 500 ± 121 | 1 367 ± 61 |  | 5.11 ± 0.02 | 0.726 |
|  |  | RNA | 609 |  | 987 ± 172 | 1 243 ± 115 |  | 4.78 ± 0.02 | 0.745 |

^1^For comparison the species frequencies were normalized to the smallest library (*n* = 37 058).

^2^Richness is the total number of OTU's in the community (i.e. the total number of independent reads within the sample; defined as aligned sequences with >97% similarity).
