# Supplementary material for: Novel Microbiological and Spatial Statistical Methods to Improve Strength of Epidemiological Evidence in a Community-Wide Waterborne Outbreak
Source: PLoS One. 2014 Aug 22;9(8):e104713. doi: 10.1371/journal.pone.0104713 (PMC4141750; doi:10.1371/journal.pone.0104713)
Supplement: Table S3 — Taxonomic affiliation of the most abundant Bacteria domain representatives. (DOC) [file pone.0104713.s003.doc]

Table S3. Taxonomic affiliation of the most abundant *Bacteria* domain representatives.

|  | The upper storage reservoir^1^ (DNA) | |  |  |  | The upper storage reservoir (RNA) | |  |
| --- | --- | --- | --- | --- | --- | --- | --- | --- |
| % | OTU code | Classification | Rank |  | % | OTU code | Classification | Rank |
| 19.2 | 001 | Comamonadaceae | family |  | 25.9 | 001 | Comamonadaceae | family |
| 2.8 | 008 | *Zoogloea* | genus |  | 9.4 | 004 | *Pseudomonas* | genus |
| 2.7 | 004 | *Pseudomonas* | genus |  | 7.5 | 002 | Methylococcaceae | family |
| 2.4 | 002 | Methylococcaceae | family |  | 5.8 | 008 | *Zoogloea* | genus |
| 2.1 | 003 | Proteobacteria | phylum |  | 3.2 | 003 | Proteobacteria | phylum |
| 1.5 | 012 | *Arcobacter* | genus |  | 1.9 | 024 | Burkholderiales | order |
|  | Tap water during contamination^2^ (DNA) | |  |  |  | Tap water during contamination (RNA) | |  |
| % | OTU code | Classification | Rank |  | % | OTU code | Classification | Rank |
| 15.2 | 001 | Comamonadaceae | family |  | 11.2 | 002 | Methylococcaceae | family |
| 2.4 | 002 | Methylococcaceae | family |  | 6.0 | 001 | Comamonadaceae | family |
| 2.4 | 012 | *Arcobacter* | genus |  | 2.9 | 008 | *Zoogloea* | genus |
| 2.3 | 003 | Proteobacteria | phylum |  | 2.7 | 006 | *Methylobacter* | genus |
| 2.1 | 045 | Betaproteobacteria | class |  | 2.4 | 005 | Methylococcaceae | family |
|  | The upper storage reservoir after cleaning (DNA) | |  |  |  | The upper storage reservoir after cleaning (RNA) | |  |
| % | OTU code | Classification | Rank |  | % | OTU code | Classification | Rank |
| 11.4 | 013 | *Pedobacter* | genus |  | 11.2 | 004 | *Pseudomonas* | genus |
| 4.2 | 003 | Proteobacteria | phylum |  | 6.1 | 003 | Proteobacteria | phylum |
| 3.7 | 074 | Bacteria | domain |  | 4.8 | 001 | Comamonadaceae | family |
| 3.3 | 004 | *Pseudomonas* | genus |  | 3.2 | 028 | Xanthomonadaceae | family |
| 3.0 | 076 | Xanthomonadaceae | family |  | 3.1 | 013 | *Pedobacter* | genus |

^1^Sampling point 5, see Fig. 1. ^2^Sampling point 7, see Fig.1.
