# Supplementary material for: Novel Microbiological and Spatial Statistical Methods to Improve Strength of Epidemiological Evidence in a Community-Wide Waterborne Outbreak
Source: PLoS One. 2014 Aug 22;9(8):e104713. doi: 10.1371/journal.pone.0104713 (PMC4141750; doi:10.1371/journal.pone.0104713)
Supplement: Table S4 — Abundance of reads associated with faecal bacteria and/or disease agents detected in the Vuorela and Toivala drinking water distribution system. (DOC) [file pone.0104713.s004.doc]

Table S4. Abundance of reads associated with faecal bacteria and/or disease agents detected in the Vuorela and Toivala drinking water distribution system.

| Class (genus/cluster) | # of reads | | | | | | | | |
| --- | --- | --- | --- | --- | --- | --- | --- | --- | --- |
|  |  | The upper storage before cleaning^1^ | |  | Tap water during contamination^2^ | |  | The upper storage after cleaning | |
|  |  | DNA | RNA |  | DNA | RNA |  | DNA | RNA |
| Bacteroidia |  |  |  |  |  |  |  |  |  |
| *Bacteroides* |  | 343 | 76 |  | 365 | 156 |  | 2 | --^3^ |
| *Parabacteroides* |  | 36 | 8 |  | 24 | 17 |  | -- | -- |
| Bacilli |  |  |  |  |  |  |  |  |  |
| *Staphylococcus* |  | 9 | 3 |  | -- | -- |  | 94 | -- |
| *Streptococcus* |  | 1 | 1 |  | 3 | 5 |  | 15 | -- |
| γ-proteobacteria |  |  |  |  |  |  |  |  |  |
| *Acinetobacter* |  | 704 | 734 |  | 316 | 261 |  | 101 | 108 |
| *Coxiella* |  | -- | -- |  | 3 | 1 |  | -- | -- |
| *Escherichia/*  *Shigella* |  | 9 | 3 |  | 8 | 14 |  | -- | -- |
| *Legionella* |  | 34 | 5 |  | 31 | 6 |  | 51 | 61 |
| *Yersinia* |  | 4 | 2 |  | 1 | -- |  | -- | -- |
| ε-proteobacteria |  |  |  |  |  |  |  |  |  |
| *Arcobacter* |  | 585 | 291 |  | 927 | 234 |  | 1 | 25 |
| Clostridia |  | 40 | 7 |  | 32 | 15 |  | *--* | *--* |

^1^Sampling point 5, see Fig. 1. ^2^Sampling point 7, see Fig.1. ^3^not detected.
