# Supplementary material for: Novel Microbiological and Spatial Statistical Methods to Improve Strength of Epidemiological Evidence in a Community-Wide Waterborne Outbreak
Source: PLoS One. 2014 Aug 22;9(8):e104713. doi: 10.1371/journal.pone.0104713 (PMC4141750; doi:10.1371/journal.pone.0104713)
Supplement: File S1 — R code for calculating the shortest direct distance and the distance via the water pipe between each inhabitant location and water pipe breakage point. (DOC) [file pone.0104713.s005.doc]

File S1. R code for calculating the shortest direct distance and the distance via the water pipe between each inhabitant location and water pipe breakage point.

origin<-c(27.xxxxx,62.xxxxx)

# extraction of the coordinates and transferring them to spatial points:

koordkk<-read.csv2("C:\\path_needs_to_be_specified_here ", header=TRUE)

xy2kk<-cbind(koordkk$EU_P, koordkk$EU_L)

xy2kk<-SpatialPoints(xy2kk)

# conversion of the .jpg figure file to a raster file, and set of coordinates

logotj=readGDAL(system.file("pictures/Vuorela_waterdistribution_KJ_070513.JPG", package="rgdal")[1])

r11j<-raster(logotj)

xmin(r11j)<-27.xxxx

xmax(r11j)<-27.xxxx

ymin(r11j)<-62.xxxx

ymax(r11j)<-62.xxxx

projection(r11j) <- "+proj=longlat +datum=WGS84 +ellps=WGS84 +towgs84=0,0,0"

#plotting the obtained figure and inhabitant points to the map

plot(r11j)

plot(xy2kk, pch=16, col="red", add=TRUE)

# change of the raster file values suitable for the calculations

r11j[r11j==255]<-NA

r11j[r11j>0]<-1

r11j[r11j==0]<-1

r11j[is.na(r11j)]<-0

logotra<-(r11j)

logotra[logotra>0]<-1

# transfer of the figure file to list of lines

pol11 <- rasterToPoints(logotra, fun=function(x){x>0})

b<-data.frame(pol11)

line<-cbind(b$x, b$y)

L1 = Line(line)

Ls1 = Lines(list(L1), ID="a")

SL1 = SpatialLines(list(Ls1))

# creation of the transition file

out <- transition(logotra, transitionFunction=mean, directions=8)

out<-geoCorrection(out, "c")

# finding the points closest in the water pipe line for each inhabitant point. Note that some of these needed to be found manually

c<-snapPointsToLines(xy2kk,SL1)

c1<-(data.frame(coordinates(c)))

c2<-SpatialPoints(c1)

# calculating the distances from the water breakage point via the water pipe line

distra7 <- costDistance(out, origin,c2)

# plotting the used paths

a2<-shortestPath(out, origin, c2)

plot(raster(a2))

# calculating the direct distance from the water breakage point for each inhabitant point

dist <- distanceFromPoints(r11j, origin)

e<-extract(dist,c)
